# Effects of Individual Essential Amino Acids on Growth Rates of Young Rats Fed a Low-Protein Diet

**DOI:** 10.3390/ani14060959

**Published:** 2024-03-20

**Authors:** Wei Liu, Tianyi Wang, Kai Zhao, Mark D. Hanigan, Xueyan Lin, Zhiyong Hu, Qiuling Hou, Yun Wang, Zhonghua Wang

**Affiliations:** 1 Ruminant Nutrition and Physiology Laboratory, College of Animal Science and Technology, Shandong Agricultural University, Tai’an 271018, China; 18706387302@163.com (W.L.); wty526@163.com (T.W.); linxueyan@sdau.edu.cn (X.L.); hzy20040111@126.com (Z.H.); houql@sdau.edu.cn (Q.H.); wangy@sdau.edu.cn (Y.W.); 2 Faculty of Engineering and Applied Science, University of Regina, Regina, SK S4S 0A2, Canada; kaizhao868@gmail.com; 3 School of Animal Sciences, Virginia Tech, Blacksburg, VA 24061, USA; mhanigan@vt.edu

**Keywords:** protein-deficit diet, amino acids supplementation, growing animal, weight gain, mTORC1

## Abstract

**Simple Summary:**

This manuscript investigates the impact of a low-protein diet supplemented with essential amino acids on the growth performance of rats, while also examining the underlying factors influencing rat growth under experimental conditions. The experimental findings substantiate the efficacy of incorporating essential amino acids into a low-protein diet to enhance growth, and repudiate the validity of the single-limiting amino acid theory used in growth models, thereby contributing to the advancement of the protein synthesis theory in growing animals. These findings offer a theoretical foundation for guiding reductions in protein feeding, resulting in reduced nitrogen excretion.

**Abstract:**

To investigate the effects of individual essential amino acids (EAA) on growth and the underlying mechanisms, EAA individually supplemented a low-protein (LP) diet fed to young rats in the present study. Treatments were an LP diet that contained 6% crude protein (CP), a high-protein (HP) diet that contained 18% CP, and 10 LP diets supplemented with individual EAA to achieve an EAA supply equal to that of the HP diet. The CP concentration of the LP diet was ascertained from the results of the first experiment, which examined the effects of dietary CP concentrations on growth rates, with CP ranging from 2% to 26%. Weight gain was increased with the supplementation of His, Ile, Lys, Thr, or Trp as compared to the LP diet (*p* < 0.05). Feed intake was greater for the His-, Lys-, and Thr-supplemented treatments as compared to the LP group (*p* < 0.05). Protein utilization efficiency was lower for the HP group than other groups (*p* < 0.01). The supplementation of Leu, Lys, and Val led to reduced protein utilization efficiency (*p* < 0.05), but the supplementation of Thr and Trp led to greater efficiency than the LP group (*p* < 0.05). Compared to the LP group, plasma urea concentrations were elevated with individual EAA supplementation, with the exception of the Thr addition. The added EAA resulted in increased concentrations of the corresponding EAA in plasma, except for Arg and Phe supplementation. The supplementation of Arg, His, Leu, Lys, and Met individually stimulated mTORC1 pathway activity (*p* < 0.05), and all EAA resulted in the decreased expression of ATF4 (*p* < 0.05). In summary, the supplementation of His, Ile, Lys, Thr, or Trp to an LP diet improved the growth performance of young rats. Responses to His and Lys additions were related to the activated mTORC1 pathway and feed intake increases. The improved growth performance resulting from the addition of a single EAA is not solely attributed to the increased plasma availability of EAA. Rather, it may be the consequence of a confluence of factors encompassing signaling pathways, the availability of amino acids, and other associated elements. The additivity of these factors results in independent responses to several EAA with no order of limitation, as is universally encoded in growth models for all production animal species.

## 1. Introduction

The metabolism of dietary amino acids (AA) and the regulation of protein synthesis in the body have garnered considerable attention owing to their profound influence on animal productivity, environmental loading, and human well-being [1,2]. Mitchell and Block (1946) proposed the single-limiting AA theory [3] as an extension of the nutrient limitation theory of Von Liebig [4]. The theory holds that protein synthesis is primarily governed by the most limiting AA (assuming adequate energy supplies), and that the supplementation of other AA will be ineffective when the most limiting AA are restricted. However, in recent years, numerous studies have presented findings that challenge this theory, particularly in investigations of the regulation of mammary lactation by AA. Basic work on the mechanisms revealed that the regulation factors involved in mammary lactation are multifactorial and additive. The independent and additive effects of essential AA (EAA) on milk protein synthesis have been observed in cattle [5,6,7]. The responses have been observed to encompass the regulation of systemic hormone levels and energy supply [8], which are believed to play a role in the regulation of visceral blood flow and nutrient uptake, among other processes, to fulfill the demands generated intracellularly [9]. This work led to a major change in the North American dairy requirement model, where milk protein responses and requirements were represented as a function of the independent effects of digested energy and five different EAA [10].

Research in growing animals has almost universally assumed that the Mitchell and Block theory is correct, and it is well documented that the supplementation of specific AA to a low-protein (LP) diet leads to enhanced growth in weanling pigs [11,12,13,14] and broiler chicks [15,16]. Numerous studies have also demonstrated that the moderate supplementation of various EAA to animal diets promotes muscle protein synthesis and improves dietary nitrogen utilization efficiencies for the branched-chain AA, Arg, Lys, Met, and Thr [17,18,19,20,21,22], and these responses are at least partially mediated by hormones [23] and signaling pathway activity [24]. However, the independent effects of individual AA on protein synthesis may differ among species and across tissues, and the sensing mechanisms have not been fully elucidated.

Because protein synthesis is energetically expensive, it makes sense that cells have mechanisms to avoid the initiation of polypeptide synthesis if the availability of any of the AA is insufficient to complete the process [25,26]. AA affect protein synthesis through at least two different signaling pathways: general control nonderepressible 2 (GCN2) and the mechanistic target of rapamycin complex 1 (mTORC1) [27,28]. The mTORC1 pathway is activated when sufficient AA are available, and phosphorylates eIF4E-binding protein 1 (4EBP1), p70 ribosomal S6 kinase (P70S6K1), and other translation regulators, thereby facilitating translation initiation and elongation, resulting in increased protein synthesis rates [29,30]. When the AA supply is deficient, the activity of the mTORC1 pathway is low and the phosphorylation level of translation regulators (e.g., 4EBP1 and P70S6K1) decreases, thereby inhibiting translation. In contrast, when AA concentrations are reduced, concentrations of the uncharged tRNA increase, and the GCN2 pathway is activated, thereby inhibiting cap-dependent translation and global protein synthesis [31], but also stimulating the increased expression of activating transcription factor 4 (ATF4), which in turn leads to the increased expression of genes involved in AA transport and non-essential AA (NEAA) biosynthesis [32,33,34]. Therefore, the expression of ATF4 tends to indicate the presence of responses to stresses such as nutritional deficiency stress and endoplasmic reticulum stress [35,36,37,38].

Several studies, in vivo and in vitro, have demonstrated that all EAA, including Arg and Gln, impact mTORC1 pathway activity [25,39,40,41,42,43], and responses to individual EAA are additive in nature [7,8]. It is possible that muscle may respond differently, but the highly conserved nature of the signaling systems argues against that possibility [44,45]. The deletion of various EAA has been shown to activate the GCN2 pathway and increase ATF4 expression in many types of cells [31,46,47]. However, no studies have systematically investigated the effects of the supplementation of each of the 10 EAA to an LP diet on growth, mTORC1 and ATF4 signaling pathways in muscle, and the relationship between growth and the mTORC1 and ATF4 signaling. Therefore, the objective of this study was to investigate the independent effects of the supplementation of 10 EAA on growth performance, mTORC1 activity, and ATF4 activity in the skeletal muscle of growing rats fed an LP diet. We hypothesize that the signaling pathways and growth will respond independently to more than one EAA, thus refuting the theory of a single AA limiting protein synthesis in growing animals. The study entails the following: (1) an examination of the growth performance, organ weight, and diet intake; (2) an assessment of the plasma biochemical indexes and AA concentrations; (3) an investigation of the mTORC1 and ATF4 pathway activity.

## 2. Materials and Methods

### 2.1. Animals, Experimental Design, and Diets

This study was conducted according to the Guide for Care and Use of Laboratory Animals. The Institutional Animal Care and Use Committee of Shandong Agricultural University approved the animal housing and handling procedures, approval number: NO. 2019-DG-0524.

The work encompassed two experiments. The first was designed to assess growth responses to varying dietary concentrations of CP so that an appropriate LP diet could be chosen. The second was designed to assess responses to individual AA when supplemented to the LP diet.

Rats were purchased from Jinan Peng Yue Experimental Animal Breeding Co., Ltd. (Jinan, China). All rats in this study were housed in individual wire-bottomed cages in a room with suitable temperature (22 ± 1 °C), humidity (50–60%), and a 12 h light/dark cycle, with ad libitum food and water intakes. Three-week-old, female Sprague-Dawley (SD) rats were acclimated to the facilities and a common diet for one week, followed by 14 days of dietary treatment. For experiment 1, 42 SD rats were randomly assigned to 7 treatment groups. The treatments diets contained CP concentrations of 2%, 6%, 10%, 14%, 18%, 22%, or 26%. Corn starch was used to balance the energy. The other components of the diets were not changed. The composition of diets is shown in Table 1.

Based on observations from the first experiment, the LP diet for the second trial was set to 6% CP, and the protein-sufficient diet (HP) was set to 18% CP, reflecting AIN-93G guidelines (Research Diets, Inc., New Brunswick, NJ, USA). Seventy-two, 3-week-old, female SD rats were randomly assigned to 12 treatment groups: the LP diet, the HP diet, and the LP diet supplemented with each of the 10 EAA. Supplementation of the EAA was set to achieve intakes of the EAA of interest equivalent to that of the 18% protein diet (see Table 2). Cornstarch was used to balance the energy. The other components of the diets were not changed. The diets were all purchased from Jiangsu Xietong Bio-engineering Co., Ltd. (Nanjing, China).

### 2.2. Sample Collection and Measure

Daily measurements of diet consumption and body weight (BW) were taken. All animals were sacrificed on day 15, between the hours of 0830 and 1000, after an overnight fast. The order in which the rats were euthanized was randomized to mitigate any potential bias resulting from time.

Blood samples were collected by ocular sampling after isoflurane anesthesia into heparinized tubes and immediately centrifuged at 1500 g for 15 min to separate plasma at a temperature of 4 °C. Plasma was subsequently stored at −20 °C for future analysis. The empty digestive tract, limbs, liver, kidneys, spleen, and carcass were collected and weighed. The right thigh muscle was collected from the identical anatomic regions of all rats, rinsed in ice-cold saline solution, immediately frozen in liquid nitrogen, and stored at −80 °C for later analyses.

An automatic biochemical analyzer (type 7020; Hitachi, Tokyo, Japan) was used to measure the plasma albumin (ALB), alanine transferase (ALT), aspartate transferase (AST), glucose (GLU), total cholesterol (CHOL), triglyceride (TG), total protein (TP), and urea with commercially available kits (Maccura Biotechnology Co., Ltd., Chengdu, China).

For AA detection, plasma samples were thawed at 4 °C, vortexed, and 10 μL was transferred to a clean 1.5 mL tube containing 10 μL of an internal standard. Forty microliters of isopropanol (plus 1% formic acid (*v*/*v*)) were added, followed by vortexing, and centrifugation at 12,000× *g* and 4 °C for 10 min to precipitate protein. Ten microliters of the supernatant were transferred to a glass HPLC vial, and derivatized using an AccQ-Fluor Reagent Kit (Waters, Milford, MA, USA) according to kit instructions. After derivatization, HPLC-MS/MS analysis was performed to determine the AA concentrations, as described by Gray et al. (2017) [48].

For Western blotting, approximately 100 mg of muscle tissue was excised from frozen thigh muscles on ice and placed in 200 μL of ice-cold cell lysis buffer (Beyotime Biotechnology, P0013J, Shanghai, China) containing protease and phosphotase inhibitors (Beyotime Biotechnology, P1046, Shanghai, China). After homogenization and ultrasound, the supernatant was obtained by centrifugation at 12,000× *g* for 10 min at 4 °C. Samples were diluted with the loading buffer (Beyotime Biotechnology, P0015L, Shanghai, China) based on protein concentration, as determined by BCA (Beyotime Biotechnology, P0009, Shanghai, China). Samples were denatured for 10 min at 100 °C, and 40 μg of sample was loaded per lane onto 4–20% gradient polyacrylamide gels (Nanjing ACE Biotechnology, F15420Gel, Nanjing, China). The separated proteins were transferred to polyvinylidene difluoride membranes (Bio-Rad, #1620261, Hercules, CA, USA) blocked with the Blocking Buffer (Beyotime Biotechnology, P0023B, Shanghai, China) for 4 h at 4 °C, followed by incubation with primary antibodies for 12 h. After removal of the primary antibody solution, the membranes were rinsed 3× with TBST for 10 min each time. The blots were probed with horseradish peroxidase-labeled secondary antibody (Beyotime Biotechnology, A0208, Shanghai, China) for 4 h at 4 °C, followed by 3 washes with TBST. Band intensity was visualized by chemiluminescence using the BeyoECL Moon Kit (Beyotime Biotechnology, P0018FM, Shanghai, China). Images of the bands were collected with a Fusion FX camera system (VILBER, Collégien, France), and quantified by ImageJ 1.53C. Phosphorylated proteins were probed first, as the abundance is lower. Following detection of the phosphorylated proteins, the blots were stripped (Beyotime Biotechnology, P0025N, Shanghai, China) and re-probed for the relative expression of the corresponding total proteins using the same procedure. The primary antibodies that were used were as follows: P-4EBP1 (phospho T37 + T46; ab278686) and 4EBP1 (ab32024), α-tubulin (ab52866) from Abcom (San Diego, CA, USA).

### 2.3. Statistics and Analysis

In the first trial, rat growth rate was regressed according to energy and protein intakes (Table 3) using a mixed model in SAS (version 9.0). In order to reduce co-linearity between protein and energy intakes, non-protein energy intake was calculated and used as the fixed effect for energy.

The statistical models used were as follows:

WG = μ + αCPI + βCPI2 + γMEI_NCP_ (1)

CPI or MEI_NCP_ = μ + αCP + βCP^2^ (2)

where WG (g) is the dependent variable (body weight gain), μ represents the intercept, CPI (g) represents the CP intake (fixed effect), MEI_NCP_ (kcal) represents the energy intake from non-protein material (fixed effect), and CP (%) represents the concentration of CP in diet DM.

Based on the results of Experiment one, a power analysis was performed to determine the δ value that could be expected to produce a significant reduction in WG as compared to the HP diet. The statistical model used was as follows:

(3) $$\delta =\frac{2\left(Z_{\alpha }+Z_{\beta }\right)\sigma }{\sqrt{n}}$$

where δ (g) is the difference between the mean value of the actual WG of rats in the HP group (WG_18_) and the theoretical WG value in the LP group (WG_LP_); *α* is the probability of type I error (*α* = 0.05); *β* is the probability of type II error (β = 0.1); *Z_α_*, *Z_β_* are the quartiles of the standard normal distribution calculated from *α* and *β*; *σ* is the overall standard deviation from the standard deviation of the Intercept in the previous statistical model.

Finally, the models derived from regressing WG_LP_ on MEI_NCP_ and CPI, and CPI on dietary CP concentrations were substituted into the WG equation and used to determine the dietary CP concentration, where the predicted WG equaled WG_LP_. The resulting dietary CP concentration was the protein concentration used for the LP diet.

A statistical analysis of growth performance, plasma biochemical indexes, plasma-free AA concentrations and Western blotting data was performed using RStudio (2022.07.2 Build 576) and R (3.6.3). One-way ANOVA was performed using the aov function. Where ANOVA results indicated a significant difference (*p* ≤ 0.05) among groups, multiple comparisons were performed using the lsd function of the PostHoc Test package, and *p*-values were corrected using the FDR method. Differences were considered significant at *p* < 0.05, and trends were considered significant at *p* < 0.10. Correlations among plasma AA concentrations and their manteltex correlation with each production index were performed using the linKET package of R. Correlations were represented as Pearson coefficients, and *p*-values were corrected using the FDR method and considered significant at *p* < 0.05 and highly significant at *p* < 0.01. The graphs were created using the ggplot2 package in the R. Multiple linear regression analysis was performed using the lm function. The model started with all of the AA, and backward elimination was conducted to reduce until all terms were significant at *p* < 0.05.

Weight data for digestive tract, limbs, liver, kidney, and spleen were all divided by BW.

Feeding efficiencies were calculated as follows:

(4) $$Feed\;utilization\;efficiency=\frac{weight\;gain}{feed\;intake}$$

(5) $$Feed\;protein\;utilization\;efficiency=\frac{weight\;gain}{crude\;protein\;intake}$$

## 3. Results and Discussion

### 3.1. Determination of Crude Protein Concentration of Low-Protein Diet

The resulting regression equations for WG vs. CPI, CPI vs. dietary CP concentration, and MEI_NCP_ vs. dietary CP concentration were as follows:

WG = 0.029 ± 0.008 × CPI^2^ + 3.19 ± 0.62 × CPI + 0.084 ± 0.013 × MEI_NCP_ − 56.9 ± 17 (*p* < 0.001, R^2^ = 0.73);

CPI = 1.83 ± 0.09 × CP + 2.55 ± 1.52 (*p* < 0.001, R^2^ = 0.93);

MEI_NCP_ =0.94 ± 0.28 × CP^2^ − 48.3 ± 9.1 × CP + 1122 ± 65 (*p* < 0.001, R^2^ = 0.80).

From these equations, the power analysis indicated that a δ value of 9.5 g would result in a statistically significant difference in WG (*p* < 0.05) when compared to rats in the HP group, with a sample size of six animals leading to the selection of the 6% CP diet as the LP diet. The HP diet was set to 18% CP according to the AIN-93G recommendations (Table 4).

### 3.2. Growth Performance

Having determined the dietary CP concentrations for the LP and HP diets, a second experiment was conducted to examine the impact of the supplementation of individual EAA to the LP diet on the growth rates of young rats. The performance results from that experiment are shown in Table 5.

Compared with the LP group, feed intake was greater for the LP + His (*p* = 0.01), LP + Lys (*p* < 0.01), and LP + Thr (*p* = 0.03) groups. The feed intake of the HP group was less than that of the LP + Arg, LP + His, LP + Ile, LP + Leu, LP + Lys, Lp + Phe, and LP + Thr groups. These responses may be related to the protein-leverage hypothesis [49], which states that, at low dietary protein concentrations, animals will increase their protein intake by increasing their feed intake, thus maintaining their body AA supply. However, the lack of such a response to the LP diet versus the HP diet (*p* = 0.27) does not support that hypothesis. It has been reported that LP diets can stimulate food intake, and the effect of individual AA on appetite is complicated and varies according to the AA and the type of imbalance among AA [25,50,51,52]. Using multiple linear regression, we found a relationship between FI and plasma concentrations of Asp, Glu, Lys, Trp, and Tau (Table 6). Clearly, the plasma AA effects on FI are not simple, with multiple AA playing a role.

**Figure 1:**
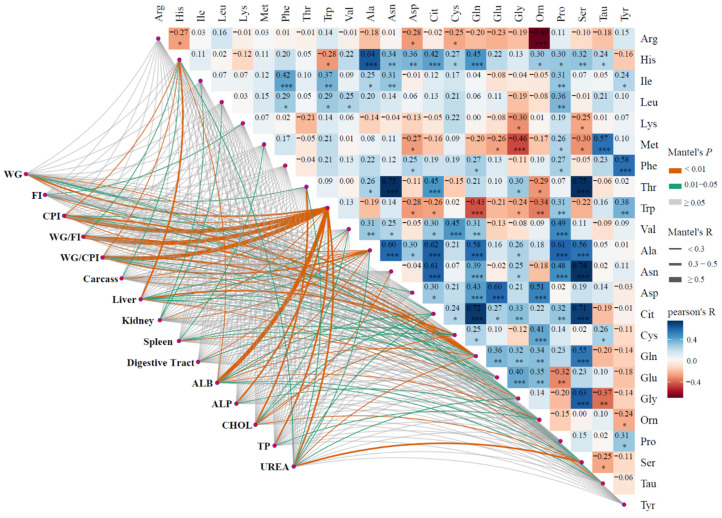
Mantel analysis between blood AA and environmental factors (growth performance or plasma biochemical indexes). WG = weight gain; FI = feed intake; CPI = crude protein intake; liver, kidney, spleen, digestive tract represent the weight percentage of weight relative to body weight, respectively; ALB = albumin; ALP = alkaline phosphatase; CHOL = total cholesterol; TP = total protein; Mantel’s *p* represents significance level of Mantel’s R between the blood AA and environmental factors. Mantel’s R represents the correlation coefficient between the blood AA and environmental factors. Pearson’s R represents the correlation coefficient between the blood AA. n = 6. * represents *p*-value of Pearson’s R < 0.05, ** represents < 0.01, *** represents < 0.001.

Animal WG was greater for the LP + His (*p* < 0.01), LP + Ile (*p* = 0.01), LP + Lys (*p* < 0.01), LP + Thr (*p* < 0.01) and LP + Trp (*p* = 0.02) groups compared to rats in the LP group. However, all LP groups exhibited lower WG compared to the HP group, suggesting that the individual contributions of His, Ile, Lys, Thr, and Trp were not the sole factors contributing to reductions in WG. The significant individual EAA responses sum to 56 g/d, which exceeds the response to HP of 29 g/d. Thus, although not tested herein, one may speculate that supplementation of a combination of His, Ile, Lys, Thr, and Trp may result in greater growth rates than those observed for the HP diet. A portion of the responses to His, Lys, and Thr were due to the increased feed intake. The correlation analysis of growth performance and plasma-free AA concentrations showed that WG was most strongly correlated with plasma Trp concentration (R = 0.44, *p* < 0.01, Figure 1), instead of the supplemented AA, which were capable of increasing WG. Multiple regression analysis showed that WG was associated with multiple AA, including Glu, Ile, Lys, Ser, Thr, and Trp (Table 6).

Feed utilization efficiency was significantly greater for the LP + His, LP + Ile, LP + Thr, and LP + Trp groups compared to the LP group (Table 5), and feed utilization efficiency was most strongly correlated with plasma Trp concentration (R = 0.73, *p* < 0.01, Figure 1). Similarly, multiple regression analysis showed that feed utilization efficiency was affected by multiple AA, including Glu, Ile, Ser, Thr, and Trp (Table 6). Studies in growing pigs showed that the addition of His or Ile facilitated feed utilization efficiency [12], which was similar to the observations of [53] in mice. The feed utilization efficiency of the rats in the HP group was significantly greater than that of the other groups. Contrary to the reduction in feed utilization efficiency, feed-protein utilization efficiency was significantly improved in all groups fed the LP diet as compared to the HP group, while the addition of Leu or Val was able to significantly reduce feed protein utilization efficiency compared to the LP group (Table 5). Feed-protein utilization efficiency was most strongly correlated with plasma Trp concentration (R = 0.73, *p* < 0.01, Figure 1). Multiple regression analysis showed that feed-protein utilization efficiency was also affected by multiple AA, including Ala, Asn, His, Leu, Thr, Trp, and Gly (Table 6).

Treatments affected carcass weight (*p* < 0.01) and the organ weight (% of BW) of the liver (*p* < 0.01), kidney (*p* < 0.01), spleen (*p* = 0.05), and digestive tract (*p* = 0.04), all relative to body weight in rats. Compared to the HP group, the liver weight increased in all other groups, while kidney and spleen weight decreased. For the LP + Met group, liver weight increased by 42% compared to the HP group (*p* = 0.01, Table 5). Cys (R = 0.38, *p* < 0.01) and His (R = 0.37, *p* < 0.01) had the largest correlation coefficients between liver weight and plasma AA concentrations (Figure 1). However, the results of multiple regression analysis showed that liver weight was associated with Arg, His, Thr, and Gly concentrations, rather than Cys (Table 6). It has been demonstrated that LP diets are able to reduce kidney weights in rats [54], and changes in the intakes of individual EAA affect liver weights [55]. There were no significant effects of treatment on the heart and limb weight.

Thus, the LP diet improved the efficiency of protein utilization but reduced the overall efficiency of feed utilization and caused a reduction in growth performance. This can all be redeemed, to some extent, by the addition of EAA, but individual EAA supplementation cannot fully recover WG to that of a HP diet. Assuming that WG responses to EAA feeding levels extend beyond the normal EAA feeding range, as observed for lactation, WG may possibly be fully rescued by the supplementation of individual EAA at a higher rate than provided in the HP diet, provided this does not cause a reduction in FI. Supplementing more than one of the responding EAA would be more likely to elicit full recovery while avoiding possible reductions in FI. Both of these hypotheses need to be tested.

The single-limiting AA theory suggests that adding only the most limiting AA can promote animal growth, and the provision of other, less limiting AA will yield no response. It also assumes that the efficiency of the conversion of absorbed EAA to product is a fixed proportion that is unaffected by the provision of other nutrients. Relative EAA limitations are calculated by subtracting maintenance costs from the predicted EAA supply and dividing the remainder by the growth yield from each unit of the metabolized EAA. The predicted growth rates allowed by the supply of each EAA are then ordered, with the lowest predicted rate designated as the 1st limiting, the 2nd lowest rate as the 2nd limiting, etc. [12,56]. The data obtained from the current study indicate that multiple EAA were simultaneously, but not exclusively, limiting WG when the rats were fed a 6% protein diet; each of five different EAA stimulated WG. This finding contradicts the single-limiting AA theory of Mitchell and Block (1946). Previous studies have also reported similar outcomes [15], where the addition of Gly, Leu, or Asp to low-protein diets promoted growth in broilers. Consequently, the single-limiting AA theory can be dismissed for growth in addition to lactation. The responses to multiple EAA also indicate that the efficiency of the conversion of each EAA to growth tissue is not a fixed value. If one EAA is shown to stimulate WG in a given diet, the slope of the response would be taken as the fixed efficiency value. If WG also responds to another EAA in the absence of a change in the first EAA, then the efficiency of conversion of the first EAA must increase to support the WG driven by provision of the second EAA. Therefore, one must also dismiss the assumption of fixed efficiencies. Efficiency also must vary as energy supply is altered, assuming that this would also affect WG, as observed for lactation [8]. The replacement of the Mitchell and Block (1946) model with one that reproduces independent responses, and the presumed additivity of responses and the variable efficiencies, should allow further progress in reducing dietary CP and likely would lead to less costly diet formulations. Given the absence of total nitrogen equilibrium across the diets, one might argue that the WG responses were due to the alleviation of a total nitrogen deficiency. However, if the observed effects are indeed attributable to total nitrogen deficiency, the introduction of any amino acid should lead to positive outcomes. The lack of responses to Arg, Leu, Met, and Val do not support this hypothesis. Leu and Val are directly transaminated, yielding NEAA, and thus the provision of NEAA in the diet could not be expected to yield a WG response unless they also stimulated the mTORC1 or GCN2 pathways.

### 3.3. Plasma Free Amino Acids Concentrations

The available data regarding the influence of dietary AA composition on AA concentrations in plasma remain limited [57]. This study aimed to examine the impact of dietary supplementation with a single EAA on plasma AA concentrations and to explore the association between variable plasma AA concentrations and WG. Plasma-free AA concentrations for each group are presented in Table 7. There was a significant increase in the plasma levels of free EAA, excluding Arg and Phe, which experienced a numerical increase when those EAA were supplemented in the diet (*p* < 0.05). In general, the concentrations during supplementation exceeded those for the HP diet, but that was not always the case. The concentrations for the LP group were also not always less than those for the HP group. Concentrations of His were consistently greater in the LP groups compared to the HP group. Furthermore, the inclusion of Leu (*p* = 0.02) and Lys (*p* = 0.02) reduced plasma His concentrations in comparison to the LP group, while the addition of Met increased His concentrations (*p* = 0.02). However, there was little correlation among the concentrations of the three AA (Figure 1). It was shown that the one-carbon metabolism involved in His is able to participate in the Met cycle [58]. The addition of Met may reduce the His metabolism, because the high Met may reduce the Met circle [59] and may reduce the need for formimino-THF in the His metabolism [60], but this still needs more study. The addition of Thr increased plasma Thr concentration nearly 57-fold compared with the LP group (*p* < 0.01), and nearly 7-fold compared with the HP group (*p* < 0.01), which is similar to previous observations [61], and the addition of Thr increased plasma Ile (*p* = 0.05) and Val (*p* < 0.01) concentrations, which is consistent with the results of a previous study [62]. The addition of Leu reduced plasma Val concentrations compared to the LP group (*p* = 0.01) and resulted in the lowest plasma concentrations of Ile among the groups, possibly due to the antagonistic effect of branched-chain AA (BCAA) [25]. It has been shown that excess Leu alters the concentrations of Val and Ile [63], whereas the addition of Val elevated the concentrations of Leu (*p* = 0.05) in the present study. As shown in Figure 1, it is noteworthy that there were strong correlations between plasma Trp concentration and several indicators: CPI (R = 0.78, *p* < 0.01), WG/FI (R = 0.73, *p* < 0.01), WG/CPI (R = 0.73, *p* < 0.01), ALB (R = 0.61, *p* < 0.01), urea (R = 0.45, *p* < 0.01), and WG (R = 0.44, *p* < 0.01). Therefore, plasma Trp concentrations may be used as a potential characteristic indicator.

Plasma NEAA concentrations are presented in Table 8. All LP-based diets increased the plasma Ala, Gln, Gly, and Ser concentrations compared to the HP group, and it has been suggested that LP diets lead to a decrease in urea production and an increase in Gln as a remedial mechanism to maintain amino pools [64]. Compared to the LP group, the addition of Met increased plasma Tau (*p* < 0.01), and decreased Gly (*p* < 0.01) and Ser (*p* < 0.01) concentrations; the addition of Phe increased plasma Tyr concentrations (*p* < 0.01); the addition of Thr increased Asn (*p* < 0.01), Ser (*p* < 0.01), and Cit (*p* < 0.01) concentrations, and resulted in the lowest blood Orn concentration among LP-based groups (approximately 44% less than the LP group); the addition of Val increased Cys concentrations (*p* < 0.01), whereas the addition of Ile and Trp did not alter any NEAA concentrations in plasma. Plasma Asp, Glu, Pro, and Orn concentrations were unaffected by treatments.

Some of the changes in NEAA can be explained mechanistically. Met can be directly metabolized to Tau [65], and the plasma concentration correlation coefficient between the two was 0.57 (*p* < 0.01, Figure 1). Serine hydroxymethyl transfer, Gly catabolism, and the de-methylation of Met are all single-carbon sources, with possible substitutions among the three relative to methyl sources and effects [66]. Consistent with the metabolism, the plasma concentrations of Ser and Gly were negatively correlated with plasma Met concentrations (−0.30, *p* = 0.04; and −0.46, *p* < 0.01, respectively; Figure 1). It may be that the high concentration of Met inhibited the Met circle, and more single-carbons are required from Gly and Ser, but this still needs more research. Tyr is produced by the dehydroxylation of Phe [67], and the correlation coefficient between the two was 0.58 (*p* < 0.01, Figure 1). Thr is the precursor of Gly or Ser [68,69], and the correlation coefficients between Thr and the other two were 0.75 (*p* < 0.01), 0.30 (*p* = 0.04), and 0.63 (*p* < 0.01), respectively (see Figure 1). However, the impact of increased protein synthesis on plasma concentrations must also be considered. The increase in WG associated with the addition of Thr increased the use of all AA for protein synthesis. One would expect this to decrease the plasma concentrations of non-supplemented AA, but this was not observed for the NEAA as compared to the LP diet. The increased net AA deposition into protein and increased WG/CPI should also decrease urea synthesis, consistent with the decline in urea concentrations for the LP + Thr treatment. Finally, decreased urea synthesis might be expected to result in reduced Cit use and Orn production [70,71]. The increase in plasma Cit concentrations with Thr additions are consistent with this latter point, but the numerical declines in Orn concentrations are not (Table 8). It has been reported that the nitrogen of Gln is mainly metabolized to Ala, ammonia, and Cit [64], and this is consistent with the positive correlation coefficients for plasma Gln concentrations with Ala and Cit concentrations of 0.58 (*p* < 0.01) and 0.72 (*p* < 0.01) (Figure 1), respectively. Finally, although not mechanistically defined, the addition of Val was shown to cause an increase in Cys concentrations in growing pigs [12], which is consistent with the correlation coefficient between plasma Val and Cys concentrations presented herein (R = 0.45, *p* < 0.01, Figure 1).

Hence, the supplementation of individual EAA to an LP diet elevates the plasma concentrations of the supplemented AA, with non-detectable changes in the other EAA. Changes in NEAA concentrations generally follow those of the EAA that are precursors, but these are not universally consistent. The relatively subtle changes in AA concentrations with individual EAA supplementation may disrupt normal amino acid metabolism, but this seems unlikely, as the changes seemingly reflect the normal distribution of plasma concentrations, and any disruption would seemingly have to affect His, Ile, Lys, Thr, or Trp.

### 3.4. Plasma Biochemical Indexes

Blood biochemical assays on plasma from all groups (Table 9) revealed that the treatments affected blood ALB (*p* < 0.01), ALP (*p* = 0.01) concentrations. Plasma ALB concentrations were significantly decreased in all LP-based groups as compared to the HP group, especially for the LP + Met group, which was less than for the LP group (*p* = 0.05, Table 9). This also suggests that albumen synthesis was decreased for the LP diets and not fully rescued by any single AA (Table 6).

The addition of Met to the LP diet may affect liver function, as previously observed [72,73]. Dietary supplementation with functional AA (e.g., Met, N-acetylcysteine, and Gly) has been shown to be beneficial in reducing or preventing oxidative stress and damage to the liver [74]. Treatments also affected plasma CHOL concentrations (*p* < 0.01), particularly the addition of Val, which resulted in the lowest plasma CHOL concentrations among all groups. Conversely, the Thr treatment resulted in the greatest plasma CHOL concentrations among groups including the HP group (*p* = 0.02). There were no effects of treatments on plasma Glucose and TG concentrations. The plasma total protein and urea concentrations were significantly greater for the HP group than for other groups, indicating that AA catabolism was reduced in the LP-based groups. Plasma urea concentrations increased to some extent in all groups (compared to LP) except for the LP + Thr group, suggesting that the additional AA are not converted to WG with the same efficiency as when added with all EAA to the HP diet [53]. It is noteworthy that the rats supplemented with Thr showed the most striking results, with the lowest liver weight and plasma urea concentrations of all groups, the highest WG and feed protein utilization efficiency except for the HP group, and the highest protein intake and plasma CHOL concentrations of all groups (Table 5 and Table 9). The reasons for the Thr results need to be further investigated, but it was the most effective at stimulating WG, and thus the nutrient use efficiency was mostly improved and urea concentrations declined. In a classical sense, the responses suggest that Thr may have been more limiting than the other EAA. A few studies have shown that the addition of Thr reduced plasma urea concentrations and improved feed utilization efficiency [62].

Although the HP diet resulted in the greatest WG, it had the lowest WG/CPI ratio and the greatest plasma urea concentrations, indicating that much more of the dietary CP was catabolized. As would be expected, the efficiency of AA utilization was greater for all LP-based treatments, and much greater for the LP + Thr and LP + Trp treatments. The latter treatments plus LP + His, LP + Ile, and LP + Lys achieved the same or better conversion efficiencies, while also gaining more BW/d than the LP diet.

### 3.5. mTORC1 and ATF4 Signaling Pathways’ Activity in Rat Muscle

**Figure 2:**
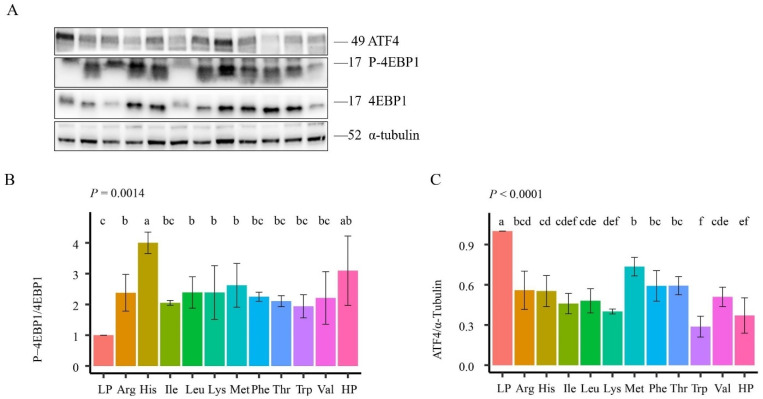
mTORC1 and ATF4 signaling pathway activity in rat muscle. (**A**) the gel images of the result of western blotting; (**B**) the phosphorylation level of 4EBP1, *P* is for the *p*-value from one-way ANOVA; (**C**), the expression level of ATF4 relative to *α*-tubulin, *P* is for the *p*-value from one-way ANOVA. n = 3. ^a–f^ Values with different superscripts differ significantly at *p* < 0.05.

Finally, to investigate the effects of EAA on the mTORC1 pathway and ATF4 (Figure 2, Supplementary Materials), the phosphorylation levels of 4EBP1 and the expression of ATF4 in the thigh muscle of rats were measured by Western blotting. The individual supplementation of Arg (*p* = 0.04), His (*p* = 0.02), Leu (*p* = 0.04), Lys (*p* = 0.04), and Met (*p* = 0.02) increased the expression levels of phosphorylated 4EBP1. In addition, there was no significant difference between His supplementation and the HP group. In fact, all supplemented EAA numerically increased the level of 4EBP1 phosphorylation as compared to the LP group. Thus, the addition of Arg, His, Leu, Lys, and Met to a 6% protein diet activated the mTORC1 pathway in the thigh muscle of rats. The addition of all individual EAA resulted in a significant decrease in ATF4 expression, and the addition of Trp inhibited ATF4 expression most significantly (*p* < 0.01). As mentioned before, ATF4 expression is a stress reaction, so all individual EAA additions alleviated the nutritional deficiency stress caused by the LP diet. It has been reported that BCAA, specifically Leu, activates the mTORC1 pathway in muscles and promotes protein synthesis [75,76,77]. Although the addition of Leu only resulted in a 10% numerical increase in WG compared to the LP group, it did stimulate the mTORC1 pathway.

The enhancement of WG with EAA supplementation is likely attributable to the activation of the mTORC1 pathway in conjunction with the suppression of ATF4 expression. Furthermore, the inclusion of His and Lys simultaneously has the potential to further augment WG. However, the precise extent to which mTORC1 contributes to this treatment necessitates further investigation, although it is evident that the activation of the mTORC1 pathway alone does not account for the observed increase in WG.

The findings herein are not consistent with the biological responses encoded in the diet formulation software used by the swine, poultry, cattle, and fish industries. The current first-limiting AA approach is based on assumptions of constant efficiencies, which imply no metabolic flexibility. The results of this work clearly demonstrate that such flexibility exists, as responses were observed with the supplementation of five different EAA into a common LP diet. Such flexibility allows for partial substitution effects. Ignoring such substitution potential likely results in greater feeding costs for the industries as each EAA must be provided to meet the target ratios for the others and the energy, regardless of cost. Representing the effects of individual EAA as additive factors in a multi-factor model captures that flexibility. For example, Thr is generally less costly to add to diets than His, Lys, or Met. Thus, one may choose to underfeed Lys or Met slightly relative to the classical requirements, and maintain performance by adding additional Thr to the diet, resulting in diet cost savings. This knowledge likely would also allow for the use of even lower dietary CP levels with an increased substitution of the economically favorable EAA, yielding a similar WG as high-protein diets and significant reductions in urinary urea excretion. The latter has additional environmental and human health benefits [1,2,78], and helps to avoid restrictions on animal numbers, as is currently happening in parts of Europe.

## 4. Conclusions

In summary, the individual supplementation of His, Ile, Lys, Thr, or Trp to an LP diet (6% protein) improved the WG of growing rats, but they did not individually restore WG to that of the HP diet. Responses to His and Lys were associated with activation of the mTORC1 pathway and feed intake increases. Furthermore, the individual supplementation of each of the EAA partially mitigated the negative effects of nutritional deficiencies on the ATF4 stress response. It is important to note that the enhanced growth performance observed with the inclusion of a single EAA cannot solely be ascribed to the heightened plasma availability of EAA. Rather, it may be the consequence of a confluence of factors encompassing signaling pathways, the availability of AA, and other associated elements.

## Figures and Tables

**Table 1:** Composition and nutrients of diets for experiment 1.

Ingredients (g/kg)	Dietary Protein Concentration						
	2%	6%	10%	14%	18%	22%	26%
Casein ^1^	22	67	112	156	200	246	291
L-Methionine ^2^	0.30	1.00	1.65	2.34	3.00	3.70	4.38
Corn starch	578	532	487	442	397	351	305
Maltodextrin	132	132	132	132	132	132	132
Sucrose	100	100	100	100	100	100	100
Cellulose	50	50	50	50	50	50	50
Soybean oil	70	70	70	70	70	70	70
Mineral mix	35	35	35	35	35	35	35
Vitamin mix	10	10	10	10	10	10	10
Choline	2.5	2.5	2.5	2.5	2.5	2.5	2.5
Butyl hydroquinone	0.014	0.014	0.014	0.014	0.014	0.014	0.014
Nutrient level (%)							
Protein	1.97	6.00	9.97	13.96	17.90	22.02	26.05
Carbohydrate	82.01	77.44	82.01	68.41	63.95	59.27	54.71
Fat	7.0	7.0	7.0	7.0	7.0	7.0	7.0
Total energy (Mcal/kg)	4.0	4.0	4.0	4.0	4.0	4.0	4.0

^1^ The CP content in casein was 88%. ^2^ the methionine content was relatively insufficient and supplemented.

**Table 2:** Composition and nutrients of diets for experiment 2.

Ingredients (g/kg)	LP	HP	LP + Val	LP + Ile	LP + Trp	LP + Thr	LP + Lys	LP + Phe	LP + Leu	LP + arg	LP + His	LP + Met
Casein ^1^	67	200	67	67	67	67	67	67	67	67	67	67
L-Valine ^2^	4.0	11.8	11.8	4.0	4.0	4.0	4.0	4.0	4.0	4.0	4.0	4.0
L-Isoleucine	3.1	9.3	3.1	9.3	3.1	3.1	3.1	3.1	3.1	3.1	3.1	3.1
L-Tryptophan	0.8	2.3	0.8	0.8	2.3	0.8	0.8	0.8	0.8	0.8	0.8	0.8
L-Threonine	2.5	7.6	2.5	2.5	2.5	7.6	2.5	2.5	2.5	2.5	2.5	2.5
L-Lysine	4.7	14.1	4.7	4.7	4.7	4.7	14.1	4.7	4.7	4.7	4.7	4.7
L-Phenylalanine	3.1	9.2	3.1	3.1	3.1	3.1	3.1	9.2	3.1	3.1	3.1	3.1
L-Leucine	5.5	16.5	5.5	5.5	5.5	5.5	5.5	5.5	16.5	5.5	5.5	5.5
L-Arginine	2.2	6.7	2.2	2.2	2.2	2.2	2.2	2.2	2.2	6.7	6.7	2.2
L-Histidine	1.7	4.9	1.7	1.7	1.7	1.7	1.7	1.7	1.7	1.7	4.9	1.7
L-Methionine	1.8	5.3	1.8	1.8	1.8	1.8	1.8	1.8	1.8	1.8	1.8	5.3
L-Cystine	1.0	3.0	1.0	1.0	1.0	1.0	1.0	1.0	1.0	1.0	1.0	1.0
Corn starch	533	398	525	526	531	527	523	526	521	538	529	529
Maltodextrin	132	132	132	132	132	132	132	132	132	132	132	132
Sucrose	100	100	100	100	100	100	100	100	100	100	100	100
Cellulose	50	50	50	50	50	50	50	50	50	50	50	50
Soybean oil	70	70	70	70	70	70	70	70	70	70	70	70
Mineral mix	35	35	35	35	35	35	35	35	35	35	35	35
Vitamin mix	10	10	10	10	10	10	10	10	10	10	10	10
Choline	2.5	2.5	2.5	2.5	2.5	2.5	2.5	2.5	2.5	2.5	2.5	2.5
Butyl hydroquinone	0.014	0.014	0.014	0.014	0.014	0.014	0.014	0.014	0.014	0.014	0.014	0.014
	Nutrients (% of DM)											
Protein	6.0	17.9	6.8	6.6	6.2	6.5	6.9	6.6	7.1	6.4	6.3	6.4
Carbohydrate	77.5	64.0	76.7	76.8	77.3	76.9	76.5	76.8	76.3	77	77.1	77.1
Fat	7.0	7.0	7.0	7.0	7.0	7.0	7.0	7.0	7.0	7.0	7.0	7.0
Total energy (Mcal/kg)	4.0	4.0	4.0	4.0	4.0	4.0	4.0	4.0	4.0	4.0	4.0	4.0

^1^ The CP content in casein was 88%. ^2^ The amounts of various AA in the table are the actual contents in the feeds.

**Table 3:** Performance of rats in response to varying dietary protein concentrations (experiment 1).

Item	Protein Concentration					
	6%	10%	14%	18%	22%	26%
WG, g	52 ± 10 ^1^	65 ± 9	60 ± 17	62 ± 9	70 ± 12	71 ± 11
Intake, g	236 ± 18	220 ± 22	192 ± 31	185 ± 11	197 ± 27	197 ± 12
CPI, g	14 ± 1	22 ± 2	27 ± 4	33 ± 2	43 ± 6	51 ± 3
MEI_DMI_, Kcal	942	879	766	739	786	788
MEI_CP_, Kcal	81	125	153	189	246	292
MEI_NCP_, Kcal	862	754	613	549	540	496

Abbreviations: WG = weight gain; CPI = crude protein intake = intake × protein concentration; MEI_DMI_ = energy from dry matter = Intake × 4; MEI_CP_ = energy from crude protein = CPI × 5.7; MEI_NCP_ = energy from non-crude protein = MEI_DMI_ − MEI_CP_. ^1^ Data style: mean ± SD. n = 6.

**Table 4:** Predicted weight gain for varying dietary protein concentrations.

CPI, g	MEI_NCP_, Kcal	WG, g	δ, g	CP
11.7	905	52.2	10.8	5%
13.5	867	53.5	9.5	6%
15.3	831	54.8	8.2	7%
17.2	797	56.1	6.9	8%
19.0	765	57.3	5.7	9%
20.8	734	58.4	4.6	10%

Abbreviations: CPI = the fitted value of crude protein intake = 1.83 × CP + 2.55, CP = crude protein; MEI_NCP_ = the fitted value of the energy from non-protein feeds = 0.94 × CP^2^ − 48.3 × CP + 1122; WG = the fitted value of the weight gain = −0.029 × CPI^2^ + 3.19 × CPI + 0.084 × MEI_NCP_ − 56.9; δ = 62.99 − WG, the fitted value of WG of the rats fed with 18% CP is 62.99 g.

**Table 5:** Effect of EAA supplementation of a low-protein diet on the growth performance of growing rats.

Item	LP	LP + Arg	LP + His	LP + Ile	LP + Leu	LP + Lys	LP + Met	LP + Phe	LP + Thr	LP + Trp	LP + Val	HP	SEM	*p*-Value
WG, g	50.3 ^e^	53.0 ^de^	62.0 ^bc^	58.7 ^bcd^	55.5 ^cde^	61.9 ^bc^	53.0 ^de^	55.3 ^cde^	66.3 ^b^	59.1 ^bcd^	52.9 ^de^	79.8 ^a^	2.3	<0.01
FI, g	224 ^def^	234 ^bcde^	250 ^ab^	239 ^abcde^	236 ^abcd^	259 ^a^	220 ^ef^	232 ^bcdef^	246 ^abc^	237 ^bcde^	228 ^cdef^	213 ^f^	5.8	<0.01
CPI, g	13.4 ^g^	15.1 ^def^	15.8 ^de^	15.9 ^cde^	16.7 ^bc^	17.9 ^b^	14.0 ^fg^	15.3 ^de^	16.0 ^cd^	14.6 ^efg^	15.4 ^de^	38.0 ^a^	0.4	<0.01
WG/FI, g/g	0.22 ^d^	0.23 ^d^	0.25 ^bc^	0.25 ^bc^	0.23 ^cd^	0.24 ^cd^	0.24 ^cd^	0.24 ^cd^	0.27 ^b^	0.25 ^bc^	0.23 ^cd^	0.38 ^a^	0.007	<0.01
WG/CPI, g/g	3.8 ^cd^	3.5 ^def^	3.9 ^abc^	3.8 ^bcd^	3.2 ^f^	3.5 ^ef^	3.8 ^bcd^	3.6 ^de^	4.1 ^a^	4.1 ^ab^	3.4 ^ef^	2.1 ^g^	0.095	<0.01
Carcass, g	54.9 ^c^	58.2 ^bc^	58.1 ^bc^	57.4 ^bc^	54.2 ^c^	63.1 ^ab^	56.5 ^c^	54.8 ^c^	59.7 ^bc^	57.3 ^bc^	53.7 ^c^	66.7 ^a^	1.9	<0.01
Liver ^1^, %	5.6 ^abc^	4.9 ^bc^	6.0 ^ab^	5.3 ^bc^	5.6 ^abc^	5.1 ^bc^	6.6 ^a^	5.4 ^bc^	4.7 ^c^	5.2 ^bc^	5.9 ^ab^	4.6 ^c^	0.003	<0.01
Kidney ^1^, %	0.87 ^ab^	0.78 ^b^	0.88 ^ab^	0.75 ^b^	0.90 ^ab^	0.77 ^b^	0.79 ^b^	0.78 ^b^	0.84 ^b^	0.82 ^b^	0.83 ^b^	1.01 ^a^	0.0004	<0.01
Spleen, %	0.22 ^a^	0.24 ^a^	0.24 ^a^	0.23 ^a^	0.22 ^a^	0.21 ^a^	0.26 ^a^	0.21 ^a^	0.22 ^a^	0.2 ^a^	0.22 ^a^	0.27 ^a^	0.0002	0.05
Heart ^1^, %	0.54	0.54	0.57	0.54	0.54	0.55	0.52	0.57	0.56	0.57	0.51	0.49	0.0003	0.68
Limbs ^1^, %	6.9	7.1	6.5	6.3	6.3	6.5	6.3	6.5	7.4	6.6	6.9	7.3	0.004	0.74
Digestive Tract ^1^, %	4.9 ^ab^	4.7 ^ab^	5.0 ^a^	4.9 ^ab^	5.2 ^a^	4.7 ^ab^	4.2 ^b^	4.7 ^ab^	4.8 ^ab^	4.8 ^ab^	4.8 ^ab^	4.6 ^ab^	0.002	0.04

Abbreviations: WG = weight gain; FI = feed intake; CPI = crude protein intake. ^a,b,c,d,e,f,g^ Values within a row with different superscripts differ significantly at *p* < 0.05. n = 6. ^1^ Organ weight divided by BW.

**Table 6:** The results of regressing performance on the plasma concentrations of individual amino acids.

Dependent Variable	Independent Variable	Estimate	SE	Variable *p*-Value	R^2^	Model *p*-Value
Weight Gain, g	Intercept	63.31	6.32	<0.001	0.59	<0.001
	Glu	0.03	0.01	0.006		
	Ile	0.15	0.06	0.020		
	Lys	0.01	0.002	0.028		
	Ser	−0.08	0.02	<0.001		
	Thr	0.01	0.001	<0.001		
	Trp	0.13	0.05	0.009		
Feed Intake, g	Intercept	245.90	9.87	<0.001	0.34	<0.001
	Asp	−0.57	0.26	0.032		
	Glu	0.08	0.04	0.026		
	Lys	0.02	0.01	0.006		
	Trp	−0.39	0.11	0.000		
	Tau	−0.32	0.11	0.005		
Weight Gain/Feed Intake,	Intercept	0.27	0.02	<0.001	0.75	<0.001
	Glu	0.0001	0.0000	0.002		
	Ile	0.0005	0.0002	0.022		
	Ser	−0.0004	0.0001	<0.001		
	Thr	0.00003	0.000005	<0.001		
	Trp	0.001	0.0002	<0.001		
Weight Gain /Protein Intake, %	Intercept	3.43	0.30	<0.001	0.75	<0.001
	Ala	0.002	0.001	<0.001		
	Asn	−0.016	0.01	0.006		
	His	0.01	0.002	0.010		
	Leu	−0.007	0.001	<0.001		
	Thr	0.0002	0.0001	0.003		
	Trp	−0.0098	0.002	<0.001		
	Gly	0.001	0.001	0.031		
Liver ^1^	Intercept	0.13	0.02	<0.001	0.43	<0.001
	Arg	−0.00024	0.0001	0.018		
	His	0.001	0.0001	<0.001		
	Thr	−0.000006	0.000003	0.025		
	Gly	−0.00008	0.00004	0.042		
Albumen	Intercept	14.76	2.02	<0.001	0.7	<0.001
	Arg	0.04	0.01	0.002		
	Cys	−0.15	0.05	0.002		
	Thr	0.001	0.0003	<0.001		
	Trp	0.11	0.01	<0.001		
	Cit	−0.08	0.02	0.001		
	Orn	0.02	0.01	0.002		

^1^ Organ weight divided by body weight. n = 6.

**Table 7:** Effect of EAA supplementation to a low-protein diet on the plasma EAA concentrations of growing rats.

EAA, μmol/L	LP	LP + Arg	LP + His	LP + Ile	LP + Leu	LP + Lys	LP + Met	LP + Phe	LP + Thr	LP + Trp	LP + Val	HP	SEM	*p*-Value
Arg	67.42	112	84	85	93	91	69	79	83	83	85	105	10	0.11
His	97.51 ^cde^	80 ^efg^	137 ^a^	107 ^bcd^	74 ^fg^	76 ^fg^	119 ^ab^	90 ^def^	104 ^bcd^	91 ^def^	113 ^bc^	63 ^g^	5.9	<0.01
Ile	23.1 ^de^	23.5 ^cde^	27.8 ^cde^	63.0 ^a^	18.3 ^e^	27.3 ^cde^	28.7 ^cd^	26.5 ^cde^	32.2 ^c^	25.8 ^cde^	29.5 ^cd^	41.6 ^b^	2.7	<0.01
Leu	125 ^d^	120 ^d^	138 ^bcd^	120 ^d^	180 ^a^	128 ^cd^	136 ^bcd^	124 ^cd^	136 ^bcd^	125 ^cd^	156 ^abc^	165 ^ab^	8.9	<0.01
Lys	726 ^d^	634 ^d^	737 ^cd^	894 ^bcd^	752 ^bcd^	1667 ^a^	849 ^bcd^	775 ^bcd^	634 ^d^	748 ^bcd^	901 ^bc^	994 ^b^	71	<0.01
Met	22.1 ^b^	22.4 ^b^	20.1 ^b^	25.7 ^b^	21.1 ^b^	27.3 ^b^	75.5 ^a^	22.8 ^b^	23.9 ^b^	20.5 ^b^	26.5 ^b^	43.1 ^b^	6.8	<0.01
Phe	68.4	73.9	76.3	82.9	71.0	72.0	80.0	84.4	73.2	73.4	79.5	83.8	4.8	0.28
Thr	59 ^c^	53 ^c^	61 ^c^	62 ^c^	50 ^c^	52 ^c^	55 ^c^	55 ^c^	3310 ^a^	53 ^c^	58 ^c^	486 ^b^	40	<0.01
Trp	36.7 ^bc^	25.4 ^c^	32.6 ^bc^	38.8 ^bc^	27.6 ^c^	34.0 ^bc^	34.6 ^bc^	31.8 ^bc^	37.6 ^bc^	43.9 ^b^	39.0 ^bc^	87.0 ^a^	4.0	<0.01
Val	24 ^d^	20 ^de^	26 ^d^	28 ^cd^	14 ^e^	26 ^d^	27 ^d^	26 ^d^	36 ^bc^	25 ^d^	145 ^a^	38 ^b^	2.5	<0.01

^a,b,c,d,e,f,g^ Values within a row with different superscripts differ significantly at *p* < 0.05. n = 6.

**Table 8:** Effect of EAA supplementation to a low-protein diet on the plasma NEAA concentrations of growing rats.

NEAA, μmol/L	LP	LP + Arg	LP + His	LP + Ile	LP + Leu	LP + Lys	LP + Met	LP + Phe	LP + Thr	LP + Trp	LP + Val	HP	SEM	*p*-Value
Ala	547 ^abc^	474 ^cd^	550 ^abc^	615 ^ab^	507 ^bcd^	473 ^cd^	593 ^abc^	517 ^abc^	648 ^a^	540 ^abc^	637 ^ab^	373 ^d^	37	<0.01
Asp	31	30	33	29	28	21	24	28	24	23	26	20	3.5	0.19
Asn	43.9 ^bcd^	37.6 ^cd^	40.3 ^bcd^	48.1 ^bc^	35.0 ^d^	43.2 ^bcd^	46.9 ^bcd^	41.8 ^bcd^	74.4 ^a^	40.2 ^bcd^	51.1 ^bcd^	43.7 ^b^	3.2	<0.01
Cys	9.1 ^b^	7.4 ^b^	9.9 ^b^	12.4 ^ab^	8.0 ^b^	10.2 ^b^	12.6 ^ab^	7.2 ^b^	7.4 ^b^	7.4 ^b^	17.0 ^a^	10.4 ^b^	1.6	<0.01
Gln	605 ^abc^	585 ^bc^	616 ^abc^	637 ^abc^	592 ^abc^	632 ^abc^	560 ^c^	580 ^bc^	696 ^ab^	599 ^abc^	714 ^a^	422 ^d^	34	<0.01
Glu	187	188	244	149	174	156	144	185	200	212	152	160	25	0.16
Gly	321 ^ab^	239 ^cd^	234 ^cd^	273 ^bc^	240 ^cd^	195 ^de^	174 ^e^	270 ^c^	322 ^ab^	330 ^a^	234 ^cd^	171 ^e^	15	<0.01
Pro	23.3 ^ab^	18.7 ^b^	22.6 ^ab^	28.1 ^ab^	22.7 ^ab^	23.8 ^ab^	25.3 ^ab^	21.0 ^b^	25.1 ^ab^	19.9 ^b^	32.6 ^a^	24.7 ^ab^	2.4	0.01
Ser	371 ^b^	313 ^cde^	313 ^cde^	350 ^bcd^	321 ^bcde^	299 ^de^	286 ^e^	322 ^bcde^	549 ^a^	348 ^bcd^	362 ^bc^	233 ^f^	16	<0.01
Tyr	11.4 ^c^	12.3 ^c^	11.8 ^c^	16.7 ^bc^	11.5 ^c^	15.3 ^c^	12.7 ^c^	29.0 ^a^	15.1 ^c^	13.1 ^c^	15.9 ^bc^	21.8 ^b^	1.8	<0.01
Tau	25.1 ^c^	21.5 ^c^	27.4 ^c^	18.8 ^c^	20.5 ^c^	17.5 ^c^	72.0 ^a^	20.8 ^c^	22.0 ^c^	20.3 ^c^	20.2 ^c^	42.1 ^b^	3.2	<0.01
Cit	76.1 ^bc^	76.3 ^bc^	81.1 ^bc^	80.9 ^bc^	74.1 ^cd^	79.3 ^bc^	72.7 ^cd^	77.0 ^bc^	96.6 ^a^	74.3 ^cd^	89.2 ^ab^	62.6 ^d^	3.8	<0.01
Orn	147	154	135	127	116	110	130	126	82	107	145	72	18	0.06

^a,b,c,d,e,f^ Values within a row with different superscripts differ significantly at *p* < 0.05. n = 6.

**Table 9:** Effect of EAA supplementation to a low-protein diet on the plasma biochemical indexes of growing rats.

Item	LP	LP + Arg	LP + His	LP + Ile	LP + Leu	LP + Lys	LP + Met	LP + Phe	LP + Thr	LP + Trp	LP + Val	HP	SEM	*p*-Value
ALB, g/L	18 ^cd^	19 ^bc^	17 ^cde^	17 ^cde^	16 ^cde^	18 ^de^	16 ^e^	18 ^bcd^	20 ^b^	18 ^bcd^	16 ^de^	25 ^a^	0.66	<0.01
ALP, U/L	641 ^ab^	805 ^a^	717 ^a^	720 ^a^	708 ^ab^	778 ^a^	640 ^ab^	755 ^a^	619 ^ab^	738 ^a^	680 ^ab^	467 ^b^	59	0.01
ALT, U/L	41	44	41	33	39	45	32	41	40	40	30	33	4.7	0.33
AST, U/L	177	144	177	135	132	164	170	141	157	143	154	115	15	0.10
Glucose, mmol/L	9.3	9.2	9.4	9.0	8.9	9.2	8.7	9.2	9.5	9.6	9.2	9.5	0.44	0.97
CHOL, mmol/L	1.5 ^bc^	1.5 ^bc^	1.7 ^b^	1.5 ^bc^	1.5 ^bc^	1.9 ^b^	1.6 ^bc^	1.7 ^bc^	2.4 ^a^	1.8 ^b^	1.2 ^c^	1.9 ^b^	0.13	<0.01
TG, mmol/L	0.75	0.64	0.83	0.67	0.93	0.80	0.66	0.76	0.68	0.92	0.67	0.65	0.10	0.45
TP, g/L	53 ^b^	55 ^b^	52 ^b^	52 ^b^	48 ^b^	54 ^b^	50 ^b^	52 ^b^	55 ^b^	49 ^b^	49 ^b^	65 ^a^	2.0	<0.01
Urea, mmol/L	2.2 ^cd^	3.4 ^bc^	3.7 ^b^	3.4 ^bc^	2.7 ^bcd^	3.0 ^bcd^	2.3 ^cd^	2.4 ^cd^	1.7 ^d^	2.4 ^cd^	3.5 ^bc^	6.8 ^a^	0.37	<0.01

Abbreviations: ALB = albumin; ALP = alkaline phosphatase; ALT = alanine transferase; AST = aspartate transferase; CHOL = total cholesterol; TG = triglyceride; TP = total protein. n = 6. ^a,b,c,d,e^ Values within a row with different superscripts differ significantly at *p* < 0.05.

## Data Availability

The data presented in this study are available in the article and Supplementary Materials.

## Supplementary Materials

The following supporting information can be downloaded at: https://www.mdpi.com/article/10.3390/ani14060959/s1, Western Blot.
