# Supplementary figures and images for: Effects of Individual Essential Amino Acids on Growth Rates of Young Rats Fed a Low-Protein Diet
Source: Animals (Basel). 2024 Mar 20;14(6):959. doi: 10.3390/ani14060959 (PMC10967486; doi:10.3390/ani14060959)

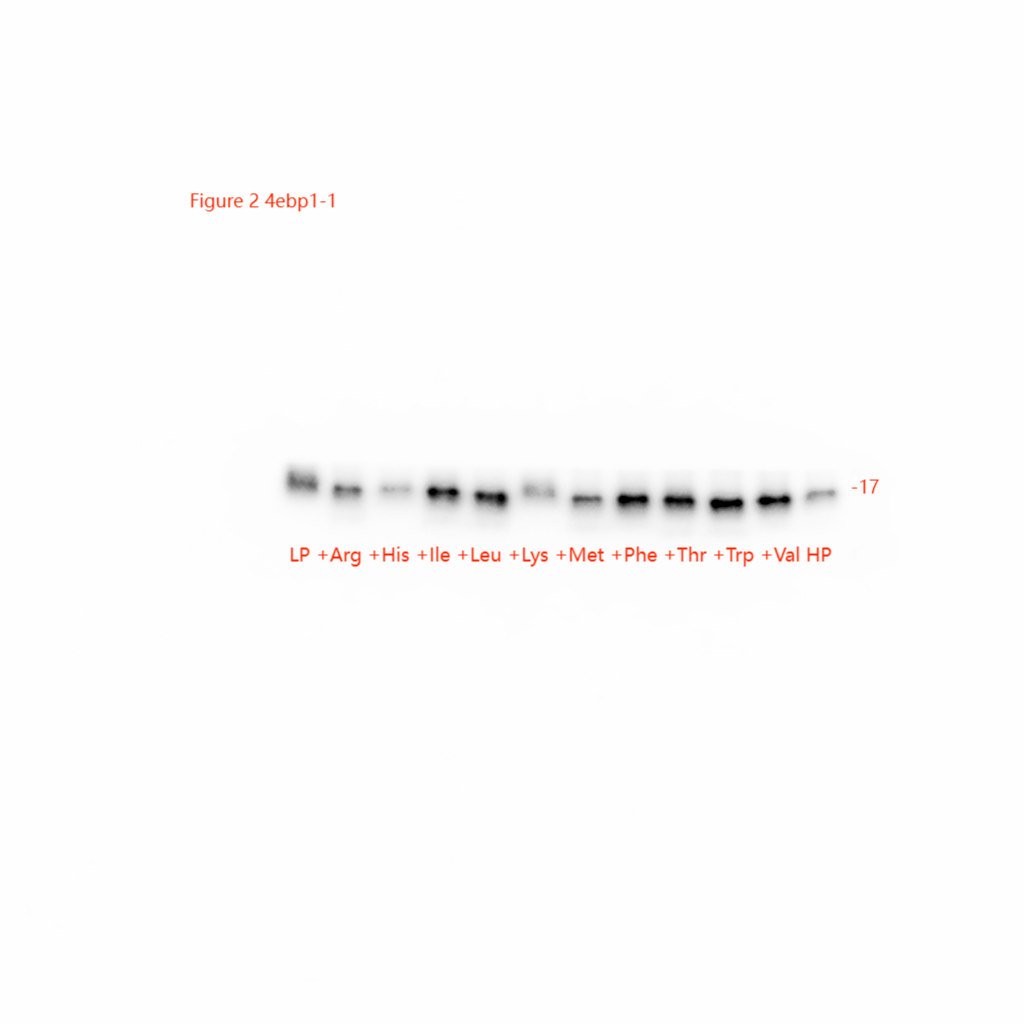

Supplement: Supplementary file 1 [file animals-14-00959-s001.zip › animals-2868708-supplementary/original blots with notes and original data/Figure 2 4ebp1-1 with note.png]

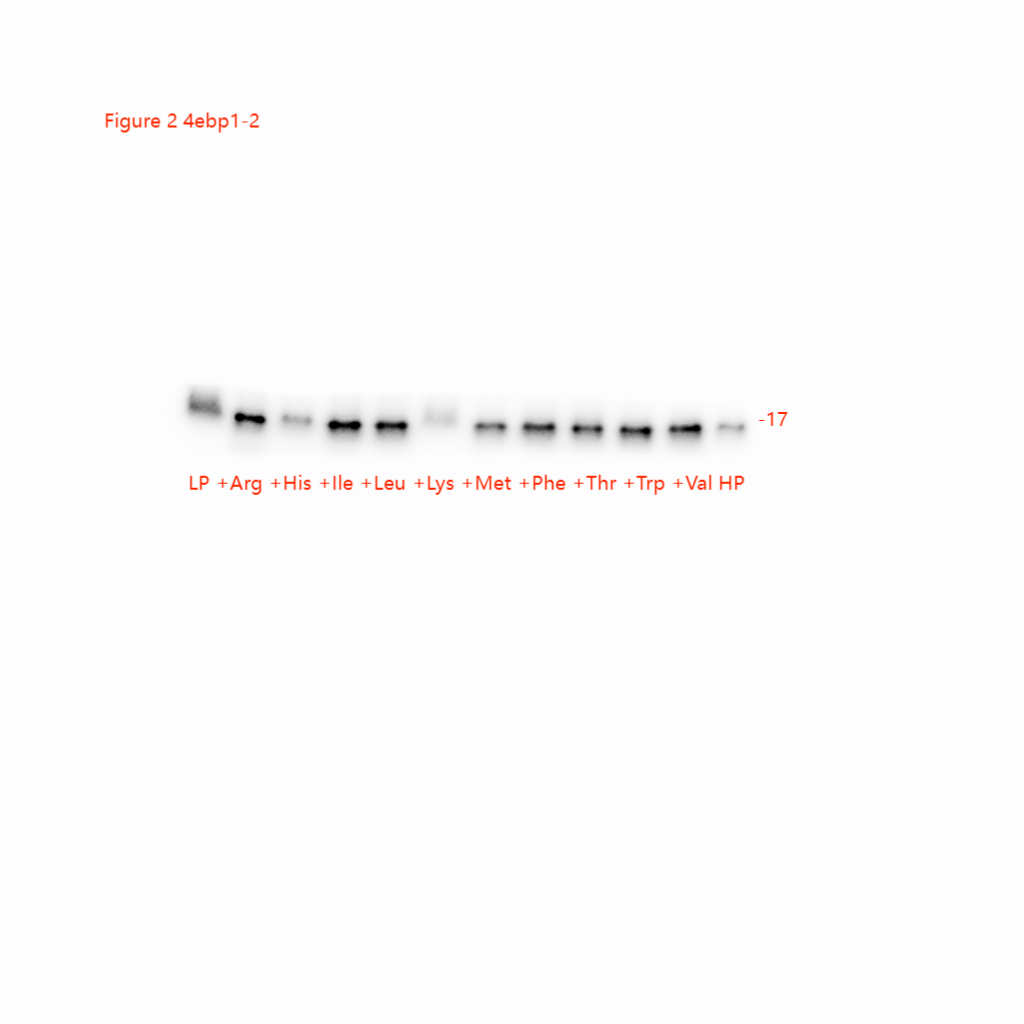

Supplement: Supplementary file 1 [file animals-14-00959-s001.zip › animals-2868708-supplementary/original blots with notes and original data/Figure 2 4ebp1-2 with note.png]

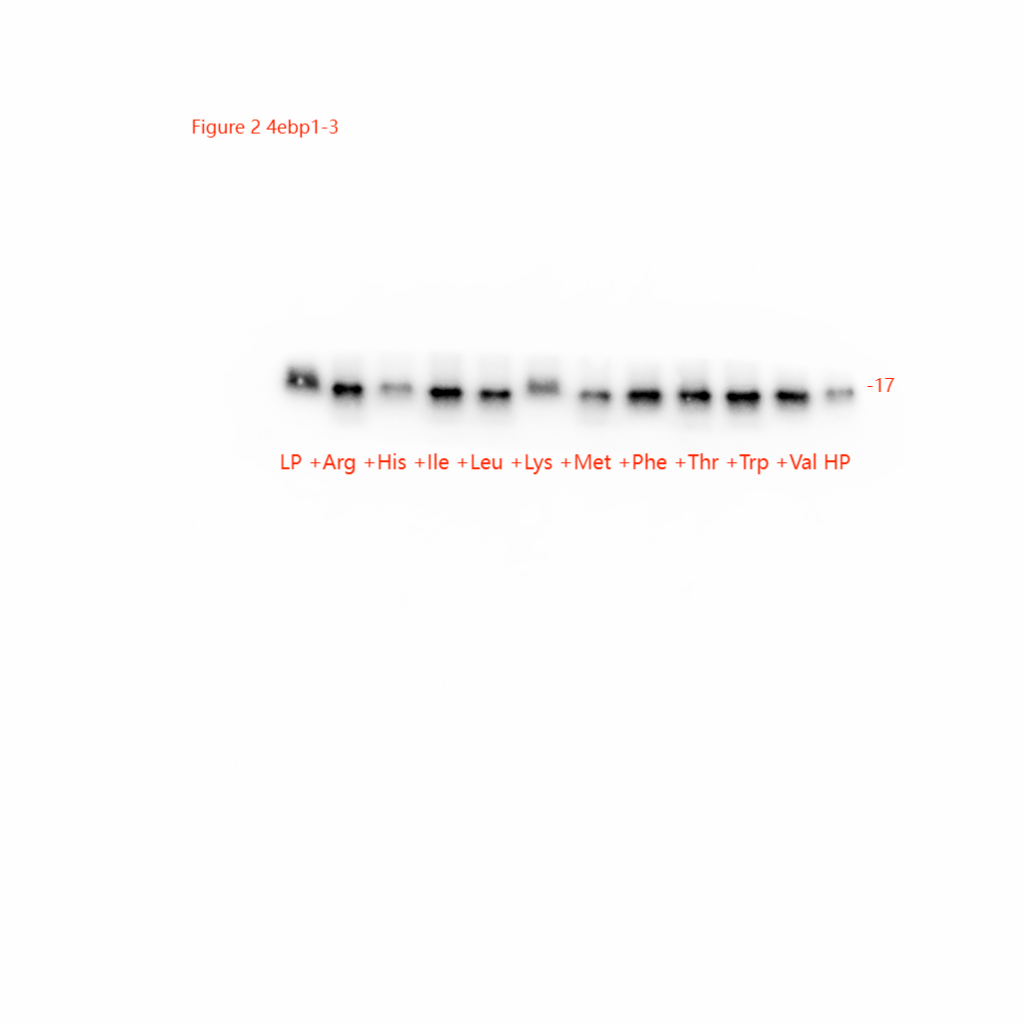

Supplement: Supplementary file 1 [file animals-14-00959-s001.zip › animals-2868708-supplementary/original blots with notes and original data/Figure 2 4ebp1-3 with note.png]

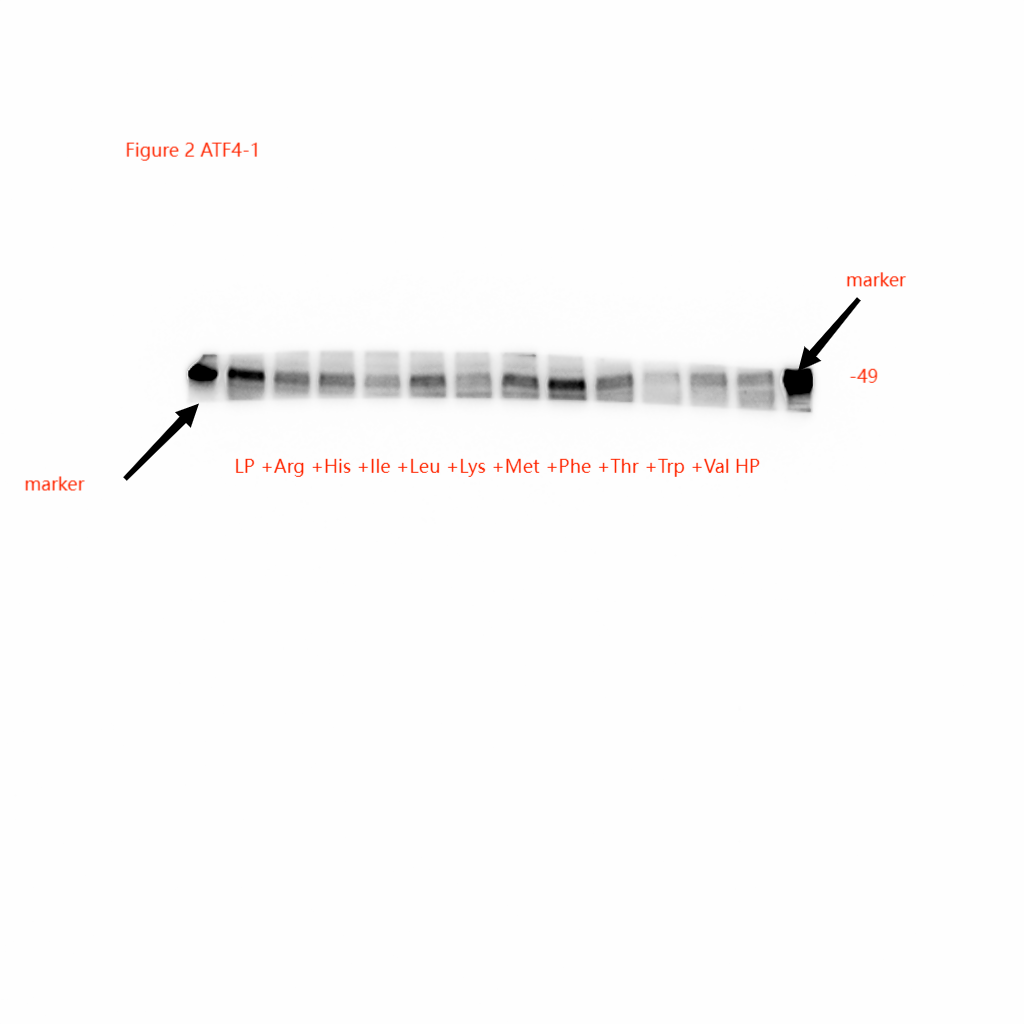

Supplement: Supplementary file 1 [file animals-14-00959-s001.zip › animals-2868708-supplementary/original blots with notes and original data/Figure 2 ATF4-1 with note.png]

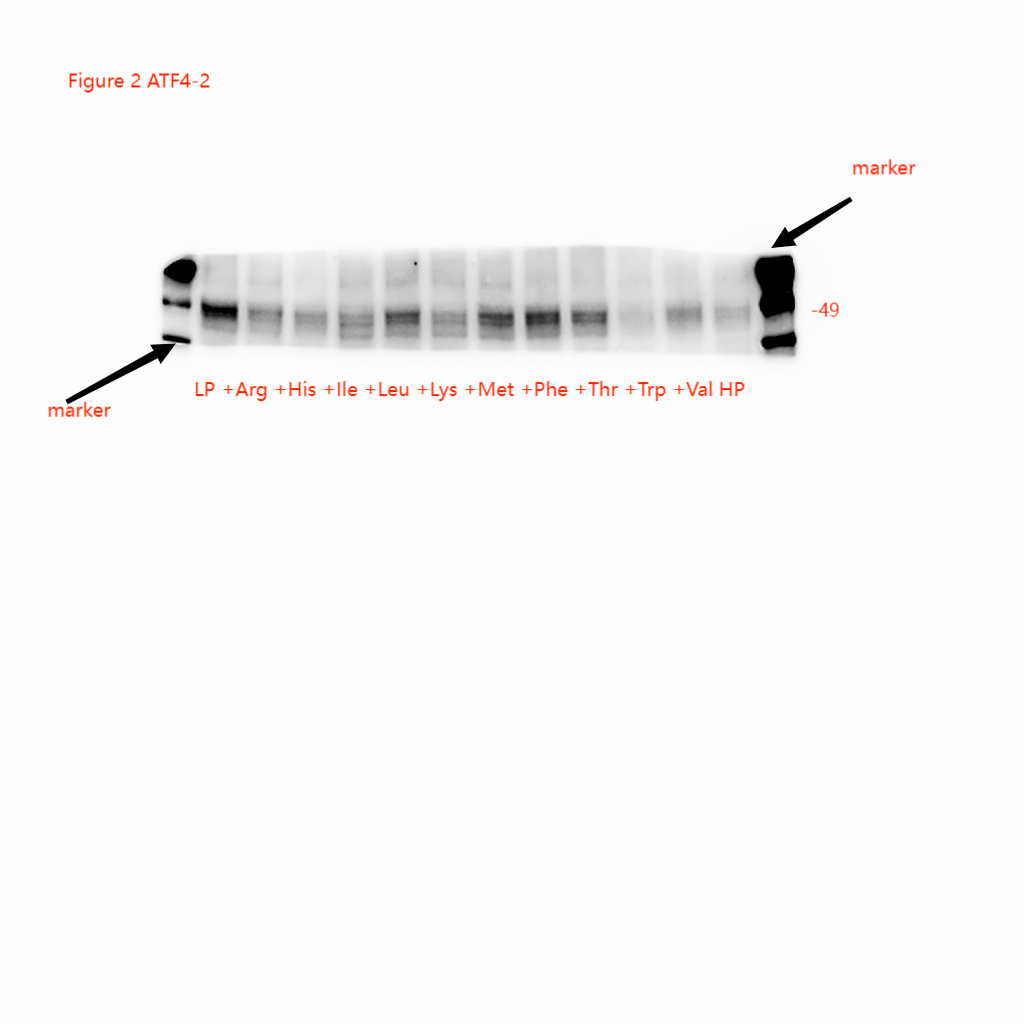

Supplement: Supplementary file 1 [file animals-14-00959-s001.zip › animals-2868708-supplementary/original blots with notes and original data/Figure 2 ATF4-2 with note.png]

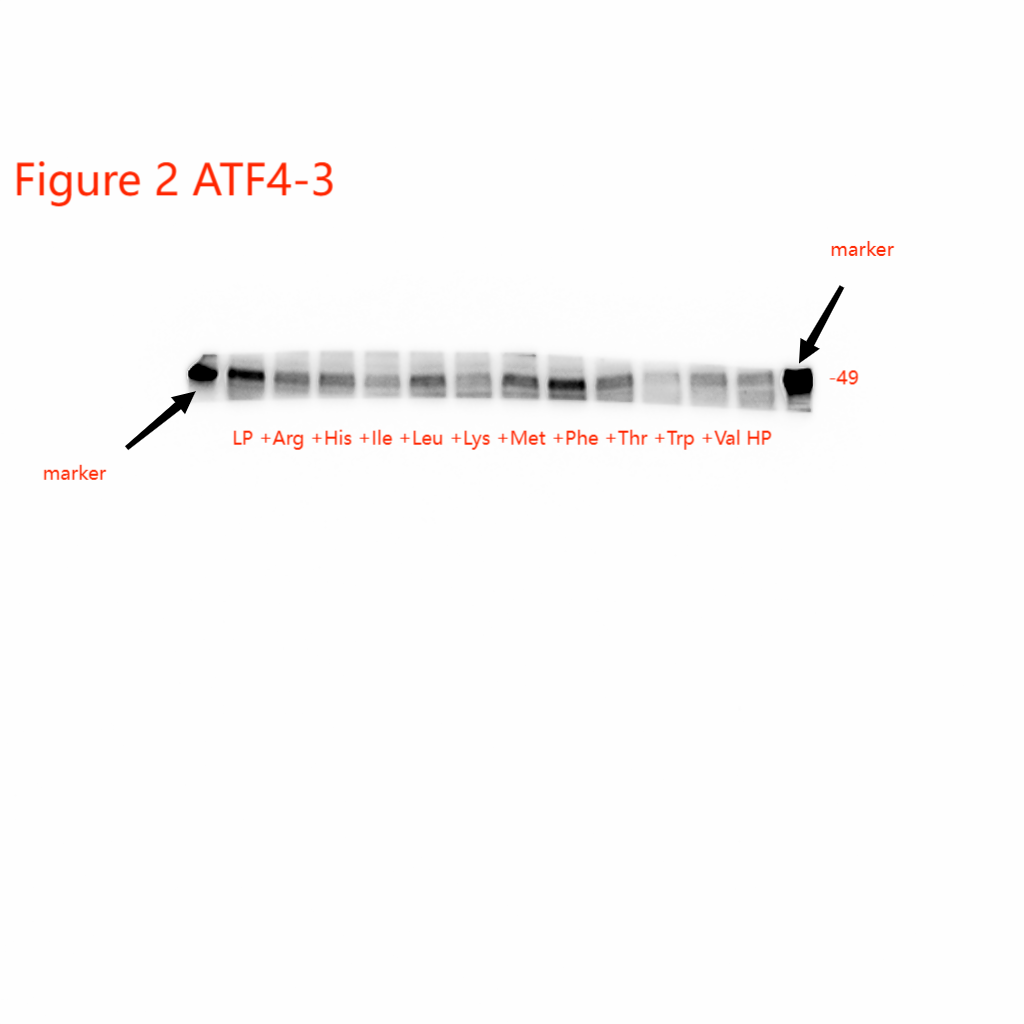

Supplement: Supplementary file 1 [file animals-14-00959-s001.zip › animals-2868708-supplementary/original blots with notes and original data/Figure 2 ATF4-3 with note.png]

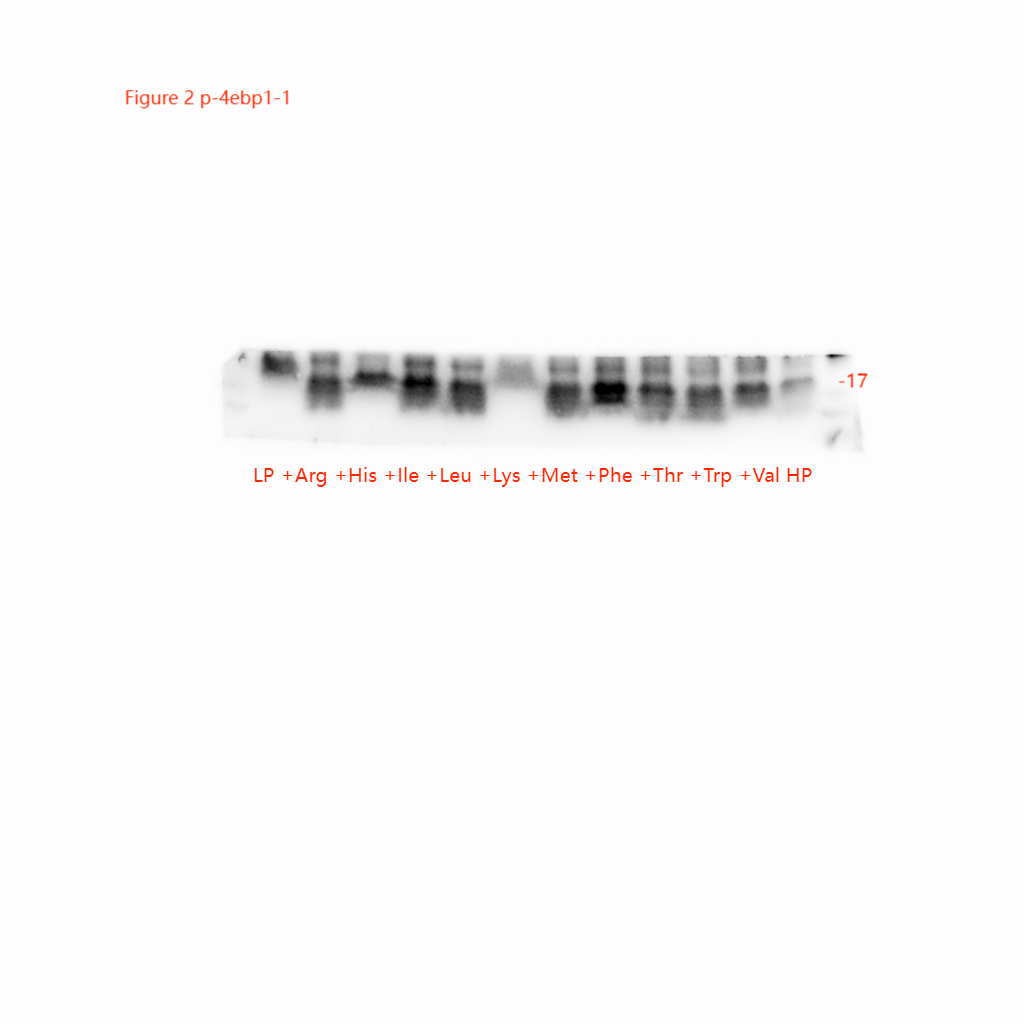

Supplement: Supplementary file 1 [file animals-14-00959-s001.zip › animals-2868708-supplementary/original blots with notes and original data/Figure 2 p-4ebp1-1 with note.png]

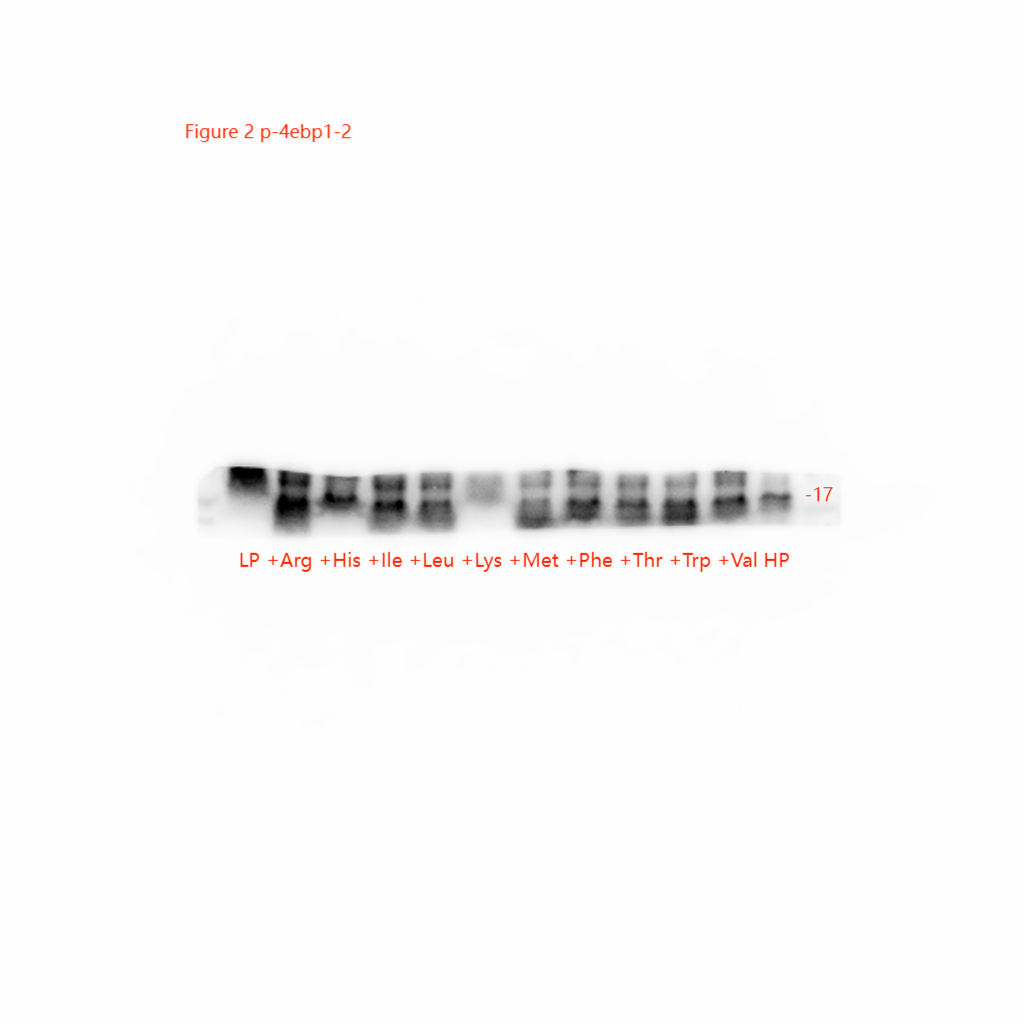

Supplement: Supplementary file 1 [file animals-14-00959-s001.zip › animals-2868708-supplementary/original blots with notes and original data/Figure 2 p-4ebp1-2 with note.png]

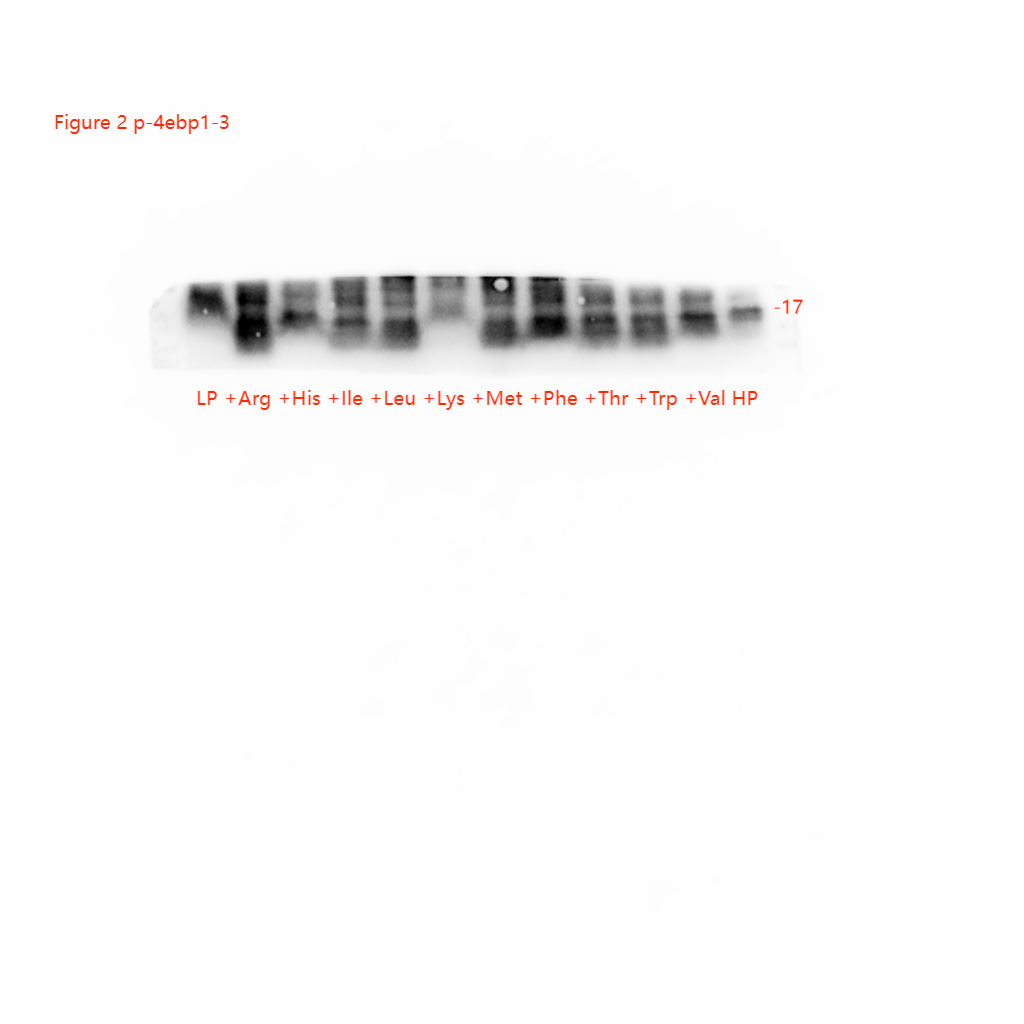

Supplement: Supplementary file 1 [file animals-14-00959-s001.zip › animals-2868708-supplementary/original blots with notes and original data/Figure 2 p-4ebp1-3 with note.png]

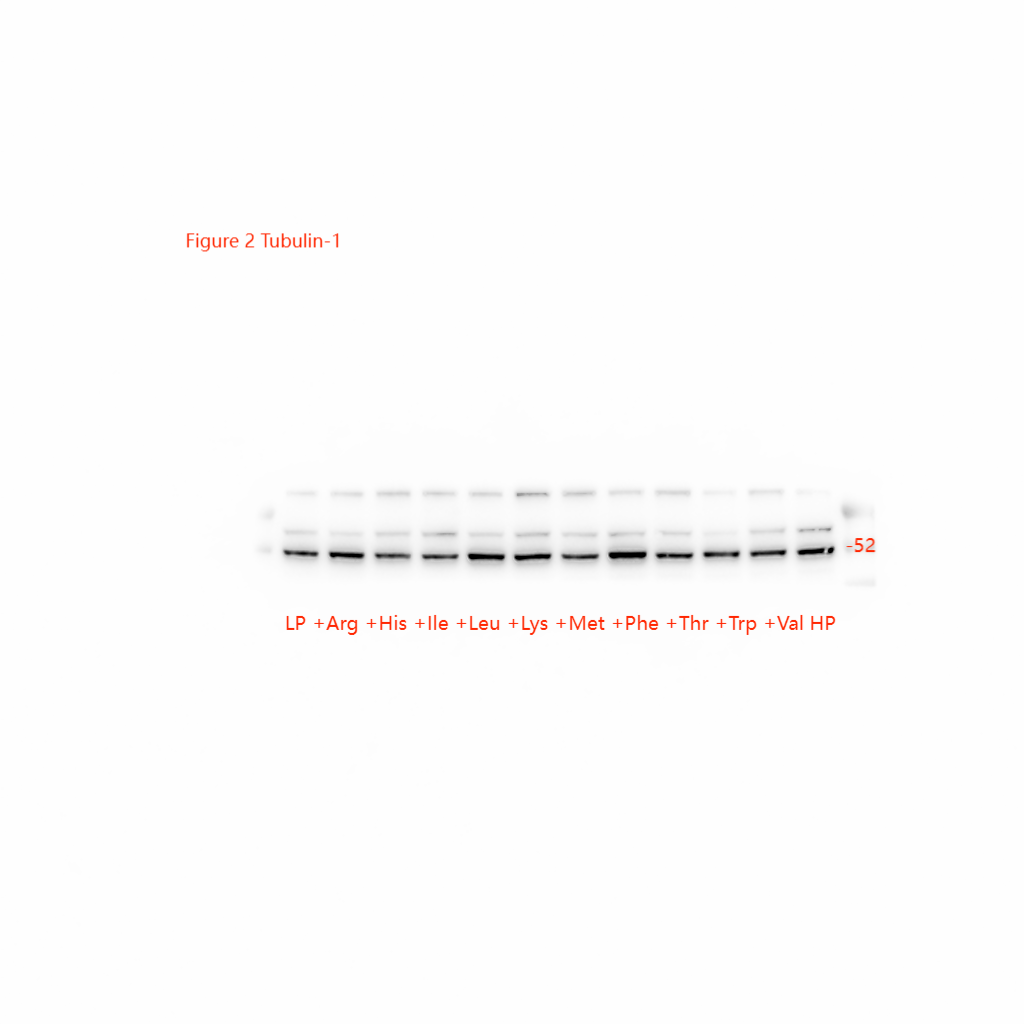

Supplement: Supplementary file 1 [file animals-14-00959-s001.zip › animals-2868708-supplementary/original blots with notes and original data/Figure 2 Tubulin-1 with note.png]

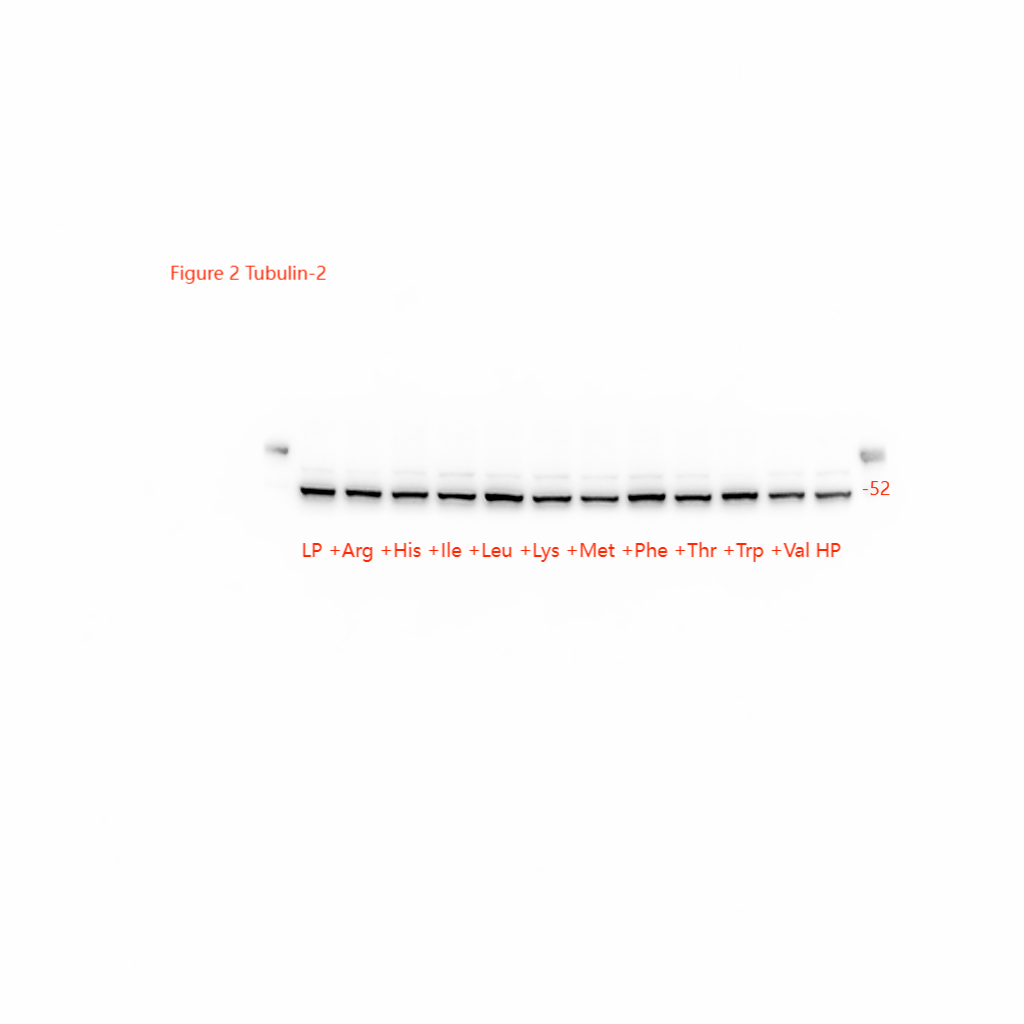

Supplement: Supplementary file 1 [file animals-14-00959-s001.zip › animals-2868708-supplementary/original blots with notes and original data/Figure 2 Tubulin-2 with note.png]

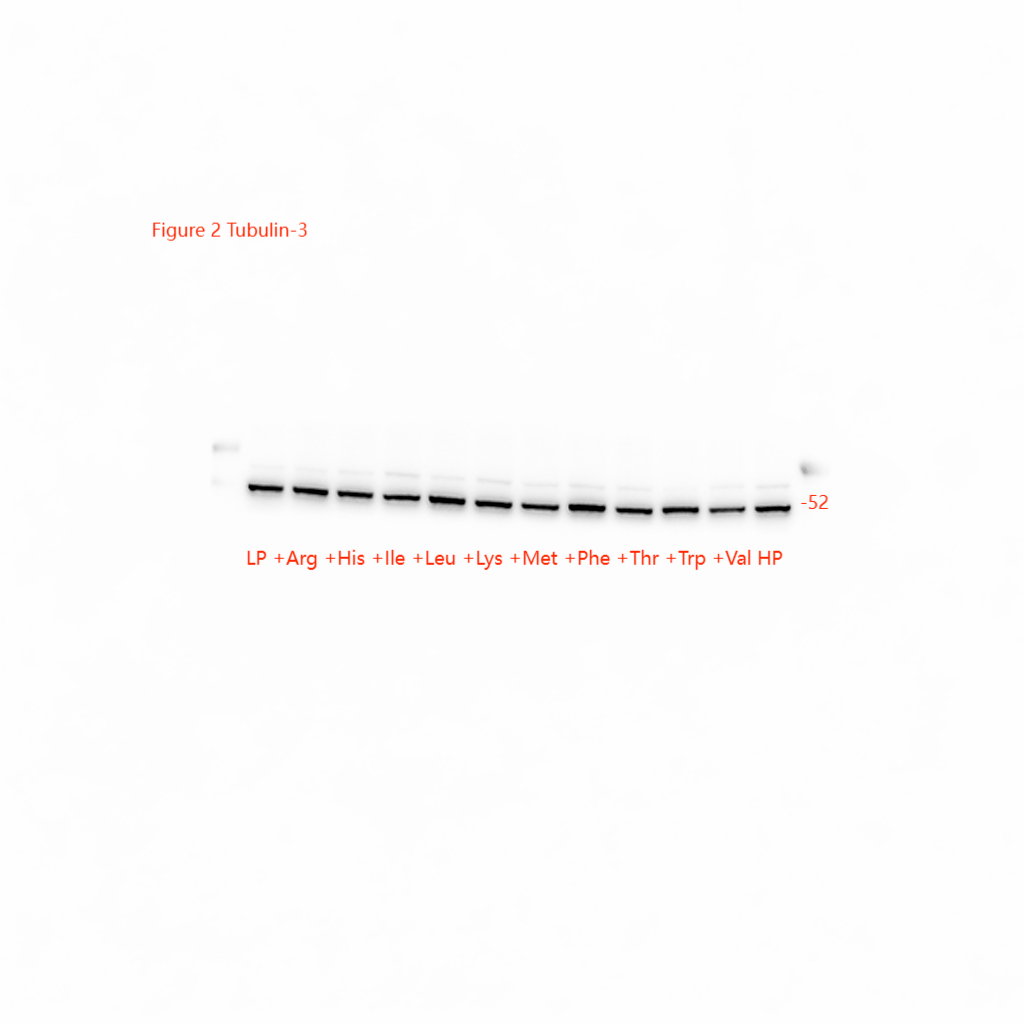

Supplement: Supplementary file 1 [file animals-14-00959-s001.zip › animals-2868708-supplementary/original blots with notes and original data/Figure 2 Tubulin-3 with note.png]
